# A supervised machine learning approach for the prediction of antioxidant activities of *Amaranthus viridis* seed

**DOI:** 10.1016/j.heliyon.2024.e24506

**Published:** 2024-01-18

**Authors:** Babatunde Olawoye, Oladapo Fisoye Fagbohun, Oyekemi Popoola-Akinola, Jide Ebenezer Taiwo Akinsola, Charles Taiwo Akanbi

**Affiliations:** a Department of Food Science and Technology, First Technical University, Ibadan, Oyo State, Nigeria; b Department of Biology, Wilmington College, Wilmington, OH, USA; c Department of Computer Science, First Technical University, Ibadan, Oyo State, Nigeria; d Department of Food Science and Technology, Obafemi Awolowo University Ile-Ife, Nigeria

**Keywords:** Data-driven models, Support vector machine, Antioxidant, Decision tree, *Amaranthus viridis*

## Abstract

This research aimed at modelling and predicting the antioxidant activities of *Amaranthus viridis* seed extract using four (4) data-driven models. Artificial Neural Network (ANN), Support Vector Machine (SVM), k-nearest Neighbour (k-NN), and Decision Tree (DT) were used as modelling algorithms for the construction of a non-linear empirical model to predict the antioxidant properties of *Amaranthus viridis* seed extract. Datasets for the modelling operation were obtained from a Box Behnken design while the hyperparameters of the ANN, SVM, k-NN and DT were determined using a 10-fold cross-validation technique. Among the Machine Learning algorithms, DT was observed to exhibit excellent performance and outperformed other Machine Learning algorithms in predicting the antioxidant activities of the seed extract, with a sensitivity of 0.867, precision of 0.928, area under the curve of 0.979, root mean square error of 0.184 and correlation coefficient of 0.9878. It was closely followed by ANN which was used to analyze and explain in detail the effect of the independent variables on the antioxidant activities of the seed extracts. This result affirmed the suitability of DT in predicting the antioxidant activities of Amaranthus viridis.

## 1 Introduction

Over the years, health degenerative diseases such as Alzheimer's, and cardiovascular disease, as well as cancer have become known due to damages in the oxidation of cellular constituents caused by reactive oxygen species. Reactive Oxygen Species (ROS) are highly reactive and unstable chemical molecules formed due to the electron acceptability of oxygen. This ROS for example, is involved in the pathogenicity of a large number of diseases [1]. The damages caused by oxidative stress which occurs due to ROS can be hindered through the consumption of foods rich in antioxidants which are derived from plant sources which in turn, led to the prevention of health-related diseases [2].

Research has demonstrated that fruits, in addition to vegetables, are natural sources of antioxidants that help to slow down the initiation of pathological diseases [3]. However, the potential of cereals/pseudo-cereals concerning their significant nutritive and health benefits is sparingly reckoned with despite its acceptability as a staple for the majority of the world populace especially people in South America [4]. On the other hand, several reports have shown that researchers are beginning to embrace the efficacy of these crops. Gupta [5] suggested the gluten-free nature of pseudo-cereals allows for incorporation into diets for those suffering from coeliac disease. Grain amaranth happens to belong to the group of pseudo-cereals gaining significant attention.

Grain amaranth, a yearly rapid-growing plant, is cultivated for its leaves and seeds. Its leaves are eaten as vegetables, incorporated in soups and salads while its seeds are used as a cereal for flour production and utilized in the preparation of muffins, bread, puddings as well as cakes [6]. A research carried out by Maurya and Arya [7] has shown that Amaranth possesses hypocholesterolemia, antitumor and antioxidant effects. The antioxidant activity of Amaranth grain is characterized by its phenolic compounds, some of which include phenolics, flavonoid, saponins and glycosides [8]. These antioxidants prevent or delay damage to cells caused by free radicals. It acts against ulcers, urinary tract inflammation, anaemia, rheumatism and asthma infection [8].

To isolate bioactive compounds from plant materials, an extraction process is required. Extraction principles dictate the methods to be used which include extraction via solvent, a process of distillation as well as getting it pressed and sublimed. Extraction via solvent has received a wide application. The raw material size, ratio of solvent-to-solid, the temperature at which it is extracted and the duration of extraction all contribute to how efficient the extraction is [9]. It is required that a choice selection is important in how the solvent is extracted. This means that how selective, how soluble, cost-effective and how safe are prerequisites for solvent selection [10]. Optimum antioxidant extraction is realizable if the above parameters are strictly adhered to. For efficiency in the extraction process, some factors capable of influencing the antioxidant properties of extracts from grain amaranths such as the type of solvent, solvent concentration, temperature, pH, the number of extraction steps, liquid-to-solid ratio and the particle size of the solute needs to be optimized. Optimizing the extraction parameters may be achieved by either empirical or statistical methods and is essential for the commercial application of the bioactive compound extraction process.

Mathematical or statistical models have widely been used for the prediction and analysis of chemical reactions or processes. Of the approaches used in mathematical modelling, data-driven modelling focuses on input-output functionality findings from experimental data sets obtained in the chemical process. One good technique that falls under data-driven modelling methods is machine learning techniques algorithms such as artificial neural network, support vector machine, k-nearest neighbours, decision trees, Naive Bayes, AdaBoost, and random forest that have found applications in the modelling, prediction and optimization of complex chemical data problems. The beauty of these machine learning techniques is there is no need for prior knowledge about experimental data and there is flexibility in determining the structure of the model as well as provision of good prediction accuracy [11]. Olawoye [12] used ANN coupled with a genetic algorithm to optimize the modification of starch, however, one constraint is the over-fitting of experimental data. SVM on the other hand, provides good prediction accuracy, owing to its tolerance to erroneous and noisy data. Its limitation is the problem encountered in the determination of optimal kernel function and hyperparameters in nonlinear modelling. Decision tree, a non-parametric machine learning algorithm, uses numerous covariates to develop prediction algorithms for dependent variables. It presents many advantages over other machine learning algorithms, in that it can be applied to different types of dependent variables which include, continuous, categorical and survival data; the high correlation of the independent variables does not influence the decision tree; finally, DT includes the most important variable influencing the dependent variables while the least important independent variables are excluded [13]. Although these machine learning algorithms have found increasing applications in chemical and biological processes, to our knowledge, their application in the prediction of the antioxidant activities of *Amaranthus viridis* seed extracts is limited. Therefore, this study aimed at predicting the antioxidant activities of *Amaranthus viridis* seed extracts using four machine learning algorithms. In this research, ANN, SVM, k-NN, and DT machine learning algorithms were evaluated and their performance measures in predicting the antioxidant activities of the seed extracts were compared.

## 2 Material and methods

The amaranth seeds used for this research were obtained from the central market of Ondo City, South-Western Nigeria. After obtaining the seeds from the market, the species of the seed was identified at the Herbarium of the Department of Botany, Obafemi Awolowo University, Ile-Ife. Afterwards, the seeds were washed thoroughly under running tap water, oven-dried at 50 °C for 9 h and milled into a fine powder with an electric grinder (Kenwood model, UK). The milled samples were subsequently packaged in an airtight container and stored at 4 °C for further analyses. Chemicals used for the analysis were obtained from Sigma-Aldrich Chemical Industries (Sigma-Aldrich, Mo, USA).

### 2.1 Extraction process

The extraction process followed the method described by Fagbohun [14]. Briefly, flour obtained from *Amaranthus viridis* seed was placed inside a conical flask containing various concentrations of methanol in the ratio of 2.5:100 (w/v). The extraction was carried out using a magnetic stirrer operating at 4000 rpm under different extraction temperatures and time as provided in the experimental design in Table 1. After extraction, the extracts obtained were passed through Whatman No. 4 filter paper and subsequently centrifuged using a BOSH centrifuge (TDL-5, UK) at 4000 rpm. The filtrate obtained was concentrated using a rotary evaporator before it was lyophilized. The lyophilized extract was stored under refrigeration before analysis.

**Table 1:** Experimental and predicted values of the antioxidant activities of extracts from Amaranthus viridis seed.

	Independent variables			Experimental responses			Predicted ANN		
Run	A	B	C	Antiradical Power (1/EC 50)	FIC (EC 50)	Frap (mg Fe(II)/100 g)	AR	FIC	FRAP
1	60	80	50	0.49	1.44	13.52	0.49	1.44	13.52
2	45	100	40	0.94	0.79	62.50	0.94	0.79	62.50
3	45	90	50	0.95	0.78	33.19	1.02	0.81	34.66
4	45	90	50	1.00	0.71	34.26	1.02	0.81	34.66
5	30	80	50	0.50	1.82	42.70	0.5	1.82	42.70
6	30	90	60	0.79	0.57	18.50	0.81	0.66	13.52
7	60	90	60	1.01	0.42	36.12	1.01	0.42	36.12
8	45	80	40	0.37	1.89	78.05	0.37	1.67	93.64
9	45	100	60	0.98	0.58	45.85	0.97	0.43	45.88
10	45	90	50	1.04	0.91	35.05	1.02	0.81	34.66
11	30	90	40	0.82	0.97	93.77	0.82	0.97	93.77
12	30	100	50	1.03	0.66	38.67	1.03	0.66	38.67
13	45	80	60	0.46	1.26	26.69	0.46	1.26	26.69
14	60	90	40	0.78	0.79	28.86	0.78	0.79	28.86
15	60	100	50	1.21	0.61	19.94	1.21	0.61	19.94
	**Predicted k-NN**			**Predicted SVM**			**Predicted DT**		
	AR	FIC	FRAP	AR	MC	FRAP	AR	FIC	FRAP
1	0.76	1.45	13.87	0.59	1.34	18.81	0.49	1.44	13.52
2	0.95	0.81	62.77	0.90	0.83	58.48	0.94	0.79	62.5
3	0.88	0.83	34.19	0.94	0.81	37.22	1	0.80	33.19
4	0.88	0.73	35.04	0.94	0.81	37.22	1	0.80	34.26
5	0.72	1.55	40.37	0.60	1.72	46.71	0.5	1.82	42.7
6	0.85	0.61	18.09	0.77	0.67	22.51	0.79	0.57	18.5
7	0.89	0.48	33.21	0.91	0.52	26.60	1.01	0.42	36.12
8	0.66	1.73	75.83	0.47	1.79	74.02	0.37	1.89	78.05
9	0.95	0.61	44.71	0.94	0.67	28.30	0.98	0.58	45.85
10	0.88	0.93	34.16	0.94	0.81	37.22	1	0.80	35.05
11	0.84	1.01	93.82	0.75	1.07	89.75	0.82	0.97	93.77
12	0.94	0.7	39.58	0.93	0.76	42.69	1.01	0.66	38.67
13	0.68	1.2	25.71	0.56	1.16	16.22	0.46	1.26	26.69
14	0.83	0.9	28.85	0.79	0.89	43.75	0.75	0.79	28.86
15	1.02	0.66	19.94	1.11	0.71	23.96	1.23	0.61	19.94

### 2.2 Experimental design using response surface methodology

The independent variables which also served as the input parameters as well as their range of values were based on the previous report of Olawoye and Gbadamosi [15]. The independent variables investigated were extraction temperature (30–60), solvent concentration (80–100 %) and extraction time (40–60 min) while the determined dependent variables (responses) were DPPH radical scavenging activity, ferrous ion chelating activity and ferrous reducing power assay. A 3 by 3 (three-level and three factors) Box-Behnken design using Statgraphics Centurion was used to obtain different combinations of the input parameters. As it can be seen in Table 1, the experimental design yielded 15 experimental runs consisting of 12 factorial designs and 3 central points, which served to minimize experimental errors.

### 2.3 Antioxidant properties of the extracts

#### 2.3.1 DPPH free radical activity

The ability of the lyophilized extract from *Amaranthus viridis* seed flour to scavenge free radicals was carried out by the method described by Fagbohun [16]. Different concentration (0.2–2.5 mg/mL) of each lyophilized extract obtained according to the experimental design in methanol was mixed with 1 mL methanolic solution containing 100 μM of DPPH radical. The extract mixture was vortexed and subsequently left to stand in the dark for 30 min. After 30 min, the absorbance of each prepared solution in different concentrations was measured at 517 nm using an ultraviolet–visible spectrophotometer (Spectrumlab 752S, England) in which methanol served as blank. Sequence to the calculation, a control sample was prepared to contain an equal volume of methanolic solution of DPPH radical as prepared for the lyophilized extract, however, the control sample contained no extract. The absorbance of the control sample was also measured at 517 nm. Ascorbic acid was used as the standard. The DPPH free radical scavenging activity was calculated and expressed in terms of antiradical power which is the inverse of EC_50_ (the concentration required to reduce initial DPPH concentration at a steady state by 50 %).

#### 2.3.2 Ferrous ion chelating activity

The ability of the *Amaranthus viridis* seed flour extract to chelate ferrous ions was determined using the modified method as described by Kadiri [17]. Different concentrations of the lyophilized extract prepared according to the experimental design were mixed with 1 mL of FeCl_2_·4H_2_O which were subsequently incubated for 5 min. Following incubation, 1 mL of ferrozine was added to initiate the reaction. After addition, the mixture was shaken vigorously using a vortex mixer and incubated for 20 min for colour development. The absorbance of the solution was measured at 562 nm using a UV–vis spectrophotometer. Ethylene-diamine tetra acetic acid was used as the control. The chelating activity of the extract was calculated using equation (1) below.

(1) $$\%Inhibition=\frac{A_{control}-A_{sample}}{A_{control}}\times 100$$

From the result, the percentage inhibition of the ferrozine-Fe^2+^ complex was obtained from which the concentration at which there was a 50 % reduction in the initial concentration of the chelating activity at steady-state (EC_50_) was calculated.

#### 2.3.3 Ferric-reducing power assay

The ability of the extract to reduce colourless Fe^3+^ ion to blue-coloured Fe^2+^ was carried out using the method of Fagbohun [18]. Briefly, a frap reagent which comprises 20 mmol/L FeCl_3_·6H_2_O, 10 mmol/L 2, 4, 6-tri-(2-pyridyl)-1, 3, 5-triazine and 300 mmol/L acetate buffer of pH 3.6 was prepared in the ratio of 1:1:10, respectively. After the preparation of the frap reagent, 1 mL of the extract solution was mixed with 1 mL of the frap reagent, vortexed and was subsequently incubated at 37 °C for 30 min to develop colour. Following incubation, the absorbance of the solution was measured at 593 nm using a UV–vis spectrophotometer. A known concentration of ferrous sulphate was used as standard and the ferric reducing power assay was expressed as the amount of the extract required to reduce 1 mmol of ferrous ion.

### 2.4 Machine learning algorithms used in the prediction of antioxidant activities

For the prediction of the antioxidant activities of the *Amaranthus viridis* seed extracts, four (4) machine learning algorithms which are the artificial neural network, k-nearest neighbour, support vector machine and decision tree were used.

#### 2.4.1 Model development and optimization using ANN coupled with genetic algorithm

**Fig. 1:**
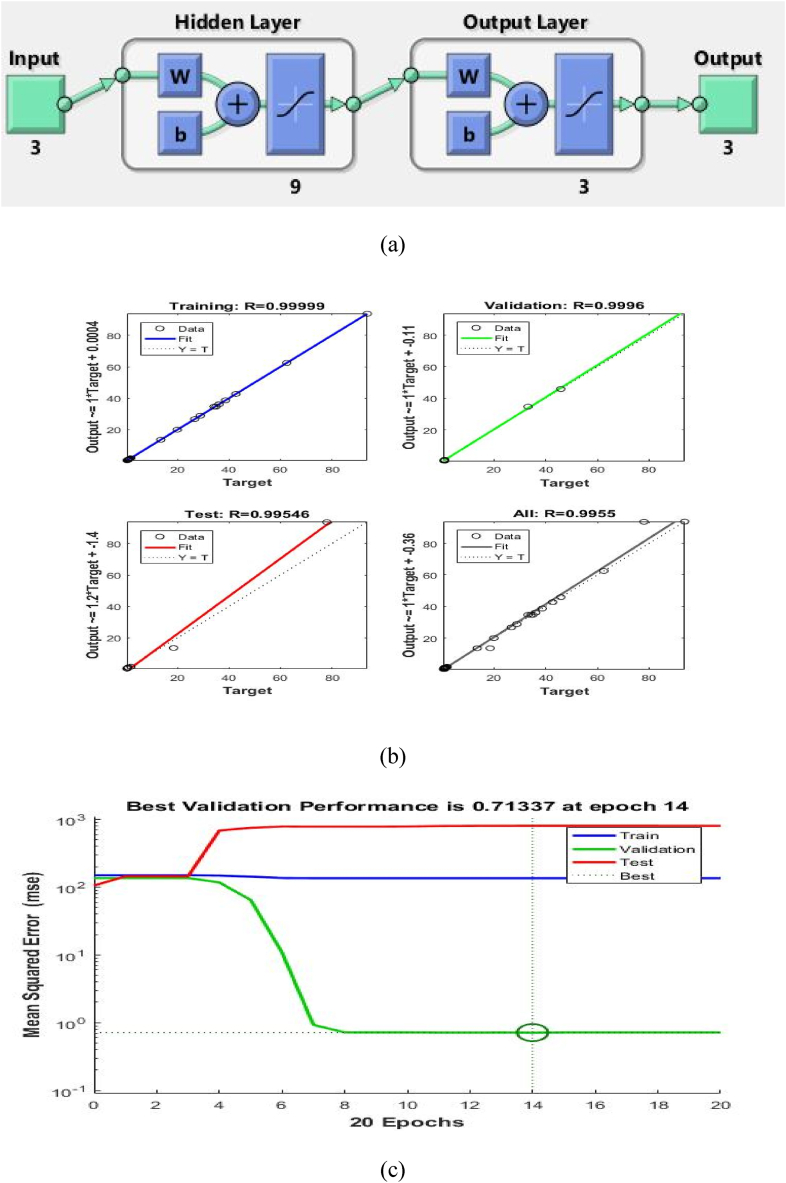
Post training: (a) neural topology; (b) performance; (c) regression analysis of generated ANN model.

The method described by Olawoye [19] was used in the modelling and optimization of the antioxidant activities of the amaranth seed extract. The results obtained from the experimental design using the Box-Behnken design were used in the development of the artificial neural network. The modelling was done using a feedforward, backpropagation multilayer perception transfer function while the training algorithm used for developing the model was the Levenberg-Marquardt backpropagation algorithm (trainlm). The reason is the most recommended among the training algorithms and the fastest. The activation functions used for the hidden and output layers were identity, logistic, tanh, exponential and sine. The modelling was done using the neural network toolbox of MATLAB R2018a (MathWorks Inc., Natick, MA, U.S.A.). The neural architecture was designed to include three input layers which are the extraction temperature, solvent concentration and extraction time; three output layers (DPPH radical scavenging activity, ferrous ion chelating activity and ferrous reducing power assay) and one hidden layer as seen in Fig. 1a. The activation function used for the hidden and output layer coupled with the optimum number of neurons for the hidden layer was determined iteratively from an algorithm with the highest coefficient of determinant (R^2^). To accurately predict the dependent variables, the network architecture was made such that 70 % of the experimental data were used for training, and 15 % for.

Testing while the validation of the experimental model was done using 15 % of the experimental data. This division of the data set was done randomly to avoid biases.

To optimize the three independent variables for maximum antioxidant activities, a genetic algorithm in which a biological evolution principle is initiated was used. Recently, GA has received much attention in that, it has a profound solution in solving regular and non-differentiable fitness functions efficiently. The GA was done using the GA tool kit in MATLAB 2018a and the properties were such that the type of population used was a double vector type while the mutation rate, selection, creation, and crossover functions were adaptive feasible, stochastic uniform, feasible population and scattered, respectively. In the course of the optimization process, all the antioxidant activities were maximized using the fitness function as given equation (2) below.

(2) $$Y=Y\left(1\right)+\frac{1}{1+Y\left(2\right)}+\frac{1}{1+Y\left(3\right)}$$

where Y was the antioxidant activities of the extracts. For the optimization, the population size, as well as the crossover fraction, were set at 50 and 0.8, respectively.

#### 2.4.2 Support vector machine

Support vector machine is a machine learning algorithm based on the Vapnik-Chervonenkis dimension theory. It is a learning algorithm that utilizes a structural risk minimization (SRM) induction principle to propound a unique solution to the experimental data set. SVM exhibits high prediction efficiency over other statistical modelling tools by recognizing a non-linear relationship between the dependent and independent variables. Also, SVR poses an advantage over other machine learning tools in that a small number of parameters are required; the kernel type as well as cost parameter C which indicates the balance in the tolerance for training errors and generalization capability. In this study, SVM was applied purposely to correlate the antioxidant activities of the *Amaranthus viridis* seed extract with the independent variables (extraction temperature, solvent concentration and extraction time). The data sets from the Box-Behnken design were used for the support vector machine (regression). The experimental data were randomly divided into two; 75 % of the data was used as a training data set while the remaining (25 %) was used as the testing data set [20]. For the modelling operation, the radial basis function kernel was selected and was characterized by equation (3) below.

(3) $$Y\left(x\right)=\sum_{n=1}^{N}w_{n}K\left(x,x_{n}\right)+w_{0}$$

where w_n_ is the model weight and K (x.x_n_) is the kernel function.

#### 2.4.3 K-nearest neighbour

k-nearest neighbour (regression), a machine learning algorithm is widely used when there is continuity in the objectives. In the application of KNN to experimental data sets, it is very important to evaluate the distance between individual training points and the objectives (response) points [21]. To model the antioxidant activities using KNN, the data set from the Box-Behnken design was used. The KNN model used a sigmoid kernel and the distance between the training points and the objectives was.

#### 2.4.4 Decision tree

Decision tree, a tree-based learning algorithm that uses regression and classification model to explain experimental outputs or responses based on categorical or numerical independent variables. The use of the decision tree in this research was because it converts data that are complex to easy to understand and informative graphical display. The algorithm ignores unnecessary nodes in the tree through a tree-pruning process and hierarchically determines the position of the independent variables by locating them at the root of the tree the most important variables. In this study, SPSS Modeler ver. 18 (SPSS Inc. Chicago IL. USA.) was used to perform the decision tree regression analysis. The data sets used for the decision tree were divided into two parts in which 75 % of the data sets were used for training while 25 % were used for testing.

### 2.5 Performance evaluation

A performance evaluation of various machine learning algorithm was evaluated on the test dataset to build the model using machine learning performance metrics that comprises ten benefit criteria such as were coefficient of correlation (R), coefficient of determinant (R^2^), adjusted R^2^, mean square error (MSE), root mean square error (RMSE), average absolute deviation (AAD) and mean absolute error (MAE). These were evaluated using equation (4 – 11) described in Table 2 below.

**Table 2:** Performance analysis of the machine learning algorithms.

Equation	Number
$R=\frac{\sum_{i=1}^{n}\left(y_{p,i}-y_{p,ave}\right)\left(y_{a,i}-y_{a,ave}\right)}{\sqrt{\left[\sum_{i=1}^{n}{\left(y_{p,i}-y_{p,ave}\right)}^{2}\right]\;\left[\sum_{i=1}^{n}{\left(y_{a,i}-y_{a,ave}\right)}^{2}\right]}}$	4
$R^{2}=1-\frac{\sum_{i=1}^{n}{\left(y_{a,i}-y_{p,i}\right)}^{2}}{\sum_{i=1}^{n}{\left(y_{p,i}-y_{a,ave}\right)}^{2}}$	5
$Adjusted\;R^{2}=1-\left[\left(1-R^{2}\right)\times \frac{n-1}{n-k-1}\right]$	6
$MSE=\frac{1}{n}\sum_{i=1}^{n}{\left(y_{p,i}-y_{a,i}\right)}^{2}$	7
$RMSE=\sqrt{\frac{1}{n}\sum_{i=1}^{n}{\left(y_{p,i}-y_{a,i}\right)}^{2}}$	8
$MAE=\frac{1}{n}\sum_{i=1}^{n}\left|\left(y_{a,i}-y_{p,i}\right)\right|$	9
$AAD=\frac{1}{n}\left(\sum_{i=1}^{n}(\frac{\left|\left(y_{a,i}-y_{p,i}\right)\right|}{y_{a,i}})\right)\times 100$	10
$SEP=\frac{RMSE}{y_{a,ave}}\times 100$	11

## 3 Result and discussion

### 3.1 Prediction of antioxidant activities of the seed extracts using ANN model

During the artificial neural network modelling, the Box-Behnken design was used to generate 15 experimental data which were randomly divided into 3 data sets. Of these data sets, 75 % was used for training, 15 % for testing and the remaining data sets (15 %) were used for the validation of the network [22]. From the experimental design table (Table 1), experimental runs 4 and 7 were used for the testing, 12 and 14 experimental runs were used for the validation of the network and the remaining runs were used for the training of the network. When modelling dependent variables using ANN, the number of neurons in the network topology must be carefully chosen to accurately predict the dependent variables. In view of this, a different number of neurons in the hidden layer were iteratively used to build the ANN model and the best was chosen based on maximum R with the result illustrated in Table 3. As could be seen in Table 3, after repeated training of the network, ten (10) neurons were selected for the hidden layer owing to its maximum R of 0.99554, 1.000, 0.9999 and 0.9979 for training, testing, and validation as well for all the data sets, respectively. Based on these criteria, a neural network architecture (Fig. 1a) was developed which consists of three (3) input layers (extraction temperature, solvent concentration, and extraction time), 9 hidden layers and 3 output layers (antiradical power, ferrous ion chelating and frap). Aside from the correlation coefficient, the weight as well as the bias which were being used for the prediction of the output (responses) data were also obtained after the training of the network and was shown in equation 12–15.

(12) $$U=\left[\begin{array}{c} 2.0095\;1.7483\;-0.8898\\ 3.0236\;1.6166\;0.0420\\ -2.4985\;0.0518\;1.0544\\ 0.4138\;1.7661\;-1.5190\\ -1.0836\;2.6456\;-0.15426\\ 1.2653\;1.0849\;2.8714\\ \;1.1721\;-1.099\;2.546\\ -2.8034\;-0..5808\;0.7823\\ 1.5037\;0.13302\;2.3079\\ -1.7953\;-2.754\;1.3219 \end{array}\right]$$

(13) $$W=\left[\begin{array}{c} 0.4201\;0.3164\;0.2524\;0.2131\;0.5653\;0.8996\;0.0911\;0.4007\;-0.8377\;-0.2081\\ 0.7015\;-0.9129\;-0.1732\;-0.3804\;-0.5281\;-1.2485\;-0..0114\;-0.1255\;1.2293\;0.1809\\ -0.8439\;0.9003\;0.7392\;-1.2845\;1.5445\;-1.4022\;-0.5121\;-1.4382\;-1.7255\;1.0288\; \end{array}\right]$$

(14) $$b_{1}=\left[\begin{array}{c} -3.3469\\ -2.4384\\ 2.0354\\ -3.0905\\ 1.5782\\ 2.0763\\ 1.6464\\ -2.6971\\ 3.1395\\ -2.5164 \end{array}\right]$$

(15) $$b_{2}=\left[\begin{array}{c} 2.8589\\ -3.7434\\ 19.1033 \end{array}\right]$$

Where U is the weight of lines that connect the neurons of the input layers to the hidden layer, W signifies the weight of lines that connect the neurons of the hidden layers to the output layer, b_1_ represents the bias value of the hidden layer neurons while b_2_ is the bias value for the output layer neurons.

**Table 3:** Effect of different neural network architecture and topology on the antioxidant activities.

Index	Net. Name	Training perf.	Test perf.	Validation perf.	Training error	Test error	Validation error	Training algorithm	Hidden activation	Output activation
**1**	MLP 3-8-3	**0.841**	**0.954**	**1.000**	**287.960**	**23.550**	**8.490**	trainlm	Sine	Exponential
**2**	MLP 3-10-3	0.975	1.000	1.000	26.880	31.720	116.600	trainlm	Logistic	Sine
**3**	MLP 3-3-3	0.473	−0.333	1.000	304.130	12.850	32.290	trainlm	Identity	Tanh
**4**	MLP 3-4-3	0.789	−0.311	1.000	294.850	93.610	63.380	trainlm	Sine	Identity
**5**	MLP 3-9-3	0.995	0.976	1.000	1.520	105.790	357.770	trainlm	Tanh	Exponential
**6**	MLP 3-9-3	0.982	0.991	1.000	286.180	13.760	85.100	trainlm	Identity	Tanh
**7**	MLP 3-9-3	1.000	0.995	1.000	0.910	1.920	0.170	trainlm	Logistic	Tanh
**8**	MLP 3-3-3	0.646	0.333	1.000	397.620	24.640	44.170	trainlm	Identity	Sine
**9**	MLP 3-9-3	0.983	1.000	0.991	53.120	84.260	57.840	trainlm	Logistic	Tanh
**10**	MLP 3-10-3	0.996	1.000	1.000	0.430	16.320	1.560	trainlm	Tanh	Sine

After the training of the neural network, the performance, as well as the regression analysis of the configuration of the neural network during training, testing and validation, are shown in Fig. 1b. The performance of MSE analysis of the neural network with respect to an increase in the number of epochs revealed that the neural network learns well and at an epoch number of (14), an MSE value of (0.7133) was obtained which indicates the best validation performance (Fig. 1c) The regression analysis of the neural network as shown in Fig b revealed the fitness of the predicted values of the antioxidant activities (output) to the actual antioxidant values (target) for the training, validation, testing and all the data sets, respectively. The predicted values of the antioxidant activities (AP, FIC and FRAP) generated using the ANN model are shown in Table 1. R-values being close to 1 is an indication of the goodness of fit and reliability of the ANN model.

### 3.2 Effect of independent variables on the antioxidant activities of the extracts

#### 3.2.1 Effect on antiradical power

**Fig. 2:**
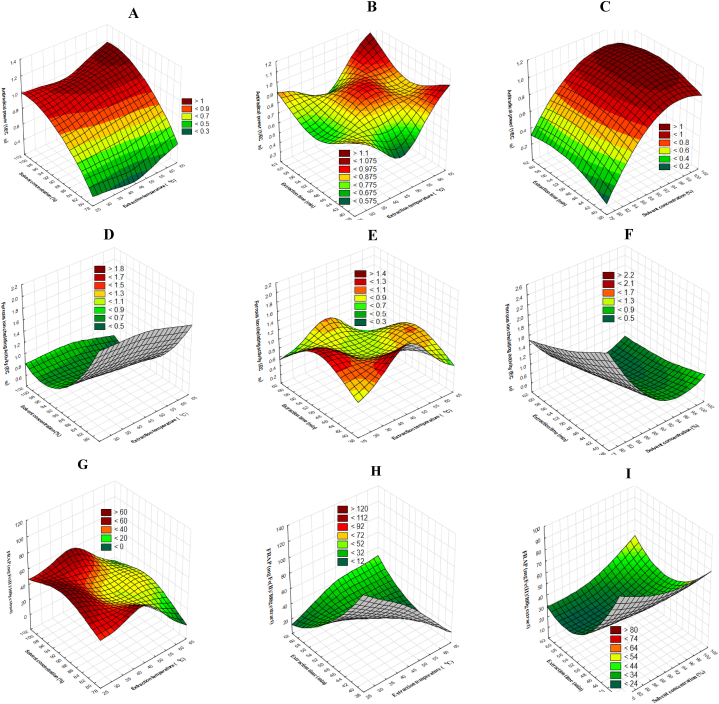
Effects of independent variables on antioxidant properties (a–c): antiradical power; (d–f) ferrous ion chelating properties Fig. 2 (g–i): Effects of independent variables on antioxidant ferric reducing power assay.

The independent effect of the extraction parameters and their interactions on the antiradical power of the *Amaranthus viridis* seed extracts using the ANN model is shown in Fig. 2a-c. The combined effect of solvent concentration and extraction temperature on the antiradical power of the extract while holding the extraction time constant is shown in Fig. 2a. Both the solvent concentration and extraction time showed a linear relationship with the antiradical power. Increasing both extraction parameters increased the antiradical power of the extracts. The extraction temperature, however, resulted in a slight decrease in the antiradical power of the extracts as it increased from 32 to 54 °C. Above 54 °C, there was a slight increase in the antiradical power. Maximum antiradical power was obtained when both the extraction temperature and the concentration of the extracting solvent were set at maximum. According to Moore and Yu [23], the higher the value of the antiradical power, the better the antioxidant activity of the extracts. In contrast to the above findings, the antiradical power of the extracts had a significant but non-linear relationship with the extraction temperature and time (Fig. 2b). In both independent variables, their initial increase led to a reduction in the antiradical power of the extracts. At maximum extraction temperature and time, a maximum antioxidant activity (antiradical power) was found. The interaction between the solvent concentration and extraction time while holding the extraction temperature constant on the antiradical power is presented in Fig. 2c. Similarly, both parameters exhibited a non-linear relationship with the antiradical power. Their increase led to an increase in the antiradical power of the extracts. Unlike the solvent concentration which resulted in a spontaneous and significant increase in the antiradical power, an increase in the antiradical power as a result of an increase in extraction time was insignificant and slow. As seen from the 3-D surface response diagram, it could be seen that maximum antiradical power could be obtained within the experimental design.

#### 3.2.2 Effect on ferrous ion chelating assay

The effects of the extraction parameters and their interaction on the ferrous ion chelating activity are shown in Fig. 2d-f. Fig. 2d represents the effect of solvent concentration and extraction temperature while the extraction time is held constant. The plot revealed that both extraction parameters had a linear effect on the chelating ability of the seed extracts. From the plot, it could be seen that an increase in solvent concentration as well as the extraction temperature resulted in a decrease in the value of the ferrous ion chelating assay and hence, the ability of the extracts to chelate ferrous ions. The result was reported in EC_50,_ that is, the concentration at which the initial concentration of ferrous ion is reduced by 50 %. The smaller the EC_50_, the higher the ferrous ion chelating ability. To achieve a minimum or least EC50 value which indicates good chelating ability, both the extraction temperature as well as solvent concentration must be at maximum. In Fig. 2e, both the extraction time and temperature had a significant but non-linear effect on the.

Ferrous ion chelating assay. An increase in the extraction temperature up to 53 °C led to a significant increase in the chelating assay which further decreased as the temperature proceeded further above 53 °C. The extraction time followed the same suit as the extraction temperature. Its increase above 57 min of extraction led to a reduction of the chelating assay. This implies that, at maximum extraction time and temperature, the ability of the extract to chelate ferrous ions increases owing to the low EC_50_ value. The ferrous ion chelating assay decreased with an increase in the extraction time as well as the solvent concentration, with the minimum value obtained when both extraction time and solvent concentration were at maximum (Fig. 2f).

#### 3.2.3 Effect on ferric reducing power assay

The extraction parameters, as well as their interaction, revealed a similar effect on the ferric reducing power assay (Fig. 2g–i). From the plot (Fig. 2g), it is evident that an increase in the extraction temperature up to 49 °C increased the FRAP. However, above that temperature, there exists a significant reduction in ferric reducing power assay. It is desirable that the extracts have a maximum FRAP and this condition can be achieved either when the extraction temperature is set at 48 °C and the solvent concentration is set at its minimum value or when the extraction temperature is set at its barest minimum when the solvent temperature is set between 86 and 97 %. Fig. 2h depicts the plot showing the interaction between extraction temperature and time while keeping the solvent concentration at a constant value (90 %). There exists a negative linear relationship between the interaction of the independent variables and ferric reducing power assay. From the plot, it could be seen that a maximum FRAP cannot be achieved within the experimental data sets. Similarly, the interaction between extraction time and solvent concentration (Fig. 2i) followed the same trend with previous FRAP plots. The increase in both values led to a reduction in the ferric reducing power assay of the extracts. For maximum ferric reducing power assay, both the extraction time and solvent concentration should be set at their barest minimum.

### 3.3 Optimizing process parameters using ANN-GA

GA has been used in various non-linear and near-linear problems owing to its ability to explore different operations for individuals making up the population size such as selection, crossover, generation, and mutation. This could be used to achieve the desired goal through the optimization of the process conditions once the neural network model is developed completely. Applying GA to the extraction process, the optimum extraction conditions were obtained after 62 iterations. The conditions were as follows: a solvent concentration of 97.02 %, extraction time of 40 min and extraction temperature of 30 °C. The predicted antioxidant activities obtained using the ANN model were 0.921 (1/Ec50) antiradical power, 0.719 Ec50 ferrous ion chelating ability and 87.31 mg Fe(II)/100 g extract. The obtained optimum extraction conditions were used in the production of extracts from the *Amaranthus viridis* seed and the response (antioxidant activities) were experimentally measured and were found to be 1.045 (1/Ec50) antiradical power, 0.704 Ec50 ferrous ion chelating ability and 89.16 mg Fe(II)/100 g extract ferric reducing power assay. The experimental values obtained commensurate well with the predicted results and hence, showed the good predictive and optimization ability of the ANN-GA model.

### 3.4 Prediction of antioxidant activities of the seed extracts using support vector machine-regression (SVM)

**Fig. 3:**
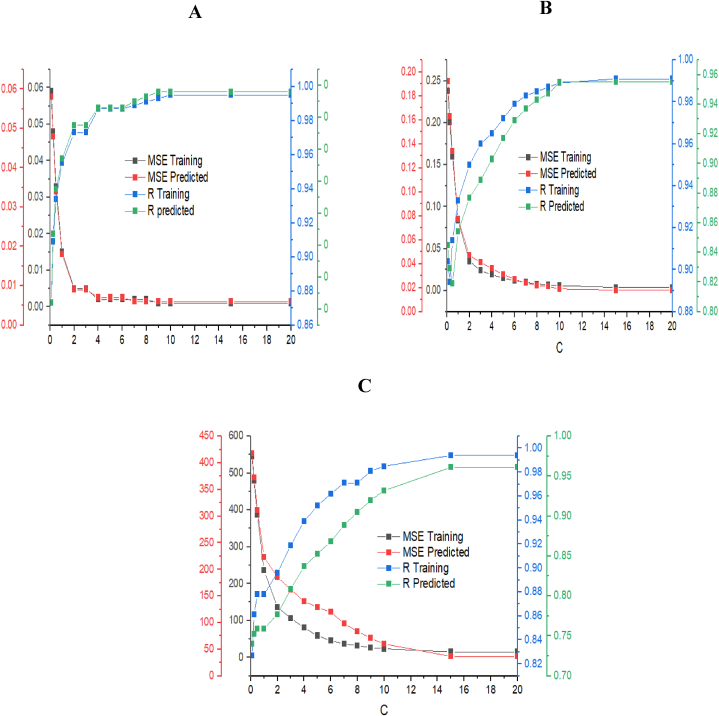
The result of various C, where ɛ = 0.01: (a) antiradical power; (b) ferrous ion chelating ability; (c) ferric reducing power assay.

Support vector machine-regression model is one of the most powerful tools used in regression analysis. The data set used in modelling the antioxidant activities of *Amaranthus viridis* seed extract was an experimental data set generated from the Box-Behnken design. The data sets were randomly divided into training and testing samples. The training constituted eleven (11) sample sizes while the rest were used in testing the SVM model. To map the original data into a higher-dimensional space, a kernel function was used and as earlier discussed in section 2.4.2, a radial basis function (RBF) was the kernel type used in the SVM-R modelling. In SVM-R, the model parameters C, y and E greatly affect the performance of the model, hence the significance of their selection and optimization to get a good SVM-R model. The constant C is the regularization constant which adjusts the ratio of the learning machine confidence interval as well as the empirical risk in the determination of data space which gives the learning machine a better generalization ability. y is a parameter that determines the width of the kernel function and needs to be optimized by the researcher. In this study, the optimization of C was carried out such that the cost of function was at a minimum. The cost of function was evaluated using the mean square of error (MSE) and correlation coefficient. To obtain the best performance of the SVM-R modelling, the training of the experimental data was done by setting y and E at a constant value of 0.333 and 0.1, respectively while C was varied between 0.1 and 20. The result as shown in Figure (3 a-c) revealed that the MSE of the trained and predicted antioxidant largely decreased and remained constant as C increases. The same was observed for the correlation coefficient which remarkably increased and remains constant as C increases. Comparing the antioxidant activities, it was found that the MSE values were at the lowest for antioxidant power, followed by ferrous ion chelating activity while the value was very high for ferric reducing power assay. The result indicated that the SVM-R algorithm is best for the prediction of antioxidant power compared to other antioxidant activities. For the best performance of the SVM-R model, the constant C was optimum at 10 which resulted in an MSE value of 0.001 and 0.006 for training and prediction as well as correlation coefficient value of 0.995 and 0.956 for training and predicted antiradical power, respectively (Fig. 3a). An optimum C value of 15 was obtained for the SVM-R modelling of the ferrous ion chelating ability of the seed extract. The corresponding MSE and R were 0.004 and 0.018; 0.991 and 0.955 for training and predicted data sets respectively (Fig. 3b). For the Ferric reducing power assay, the obtained C was 15, corresponding to an MSE and R-value of 15.16 and 36.74; 0.994 and 0.961 for training and predicting data sets respectively (Fig. 3c). The errors, as well as the correlation coefficient obtained, were considered acceptable for the continuity of the SVM modelling of the antioxidant activities of the seed extracts. It was also found that the number of the support vectors obtained during the SVM-R modelling of the antioxidant activities after optimization of C were 8, 7 and 9 with zero bounded vector for antioxidant power, ferrous ion chelating activity and ferric reducing power assay, respectively. Table 1 shows the predicted antioxidant activities obtained using SVM model with their corresponding R^2^ being 0.9791, 0.9813 and 0.8690 for antioxidant power, ferrous ion chelating activity and ferric reducing power assay, respectively. The closeness of the coefficient of determinant to 1 indicates the predictability as well as the goodness of fit of the SVM-R model.

### 3.5 Prediction of antioxidant activities of the seed extracts using k-NN

K-nearest neighbour is a non-parametric technique used in data modelling and optimization. Like the other machine learning algorithms, datasets obtained from the Box-Behnken design were in the modelling of the antioxidant activities of the seed extracts. The datasets were randomly divided into two in which 75 % of the datasets were used in the training of the model while 25 % were used in testing the experimental model. The training datasets were used in model development while the validation of the model was done using the testing datasets. The performance of the k-NN algorithm is dependent on the number of neighbour samples (n_neighbour), therefore, different values of neighbour samples ranging from 1 to 10 neighbours were used in training the dataset. The number of neighbours selected was based on the neighbours with the highest coefficient of.

**Fig. 4:**
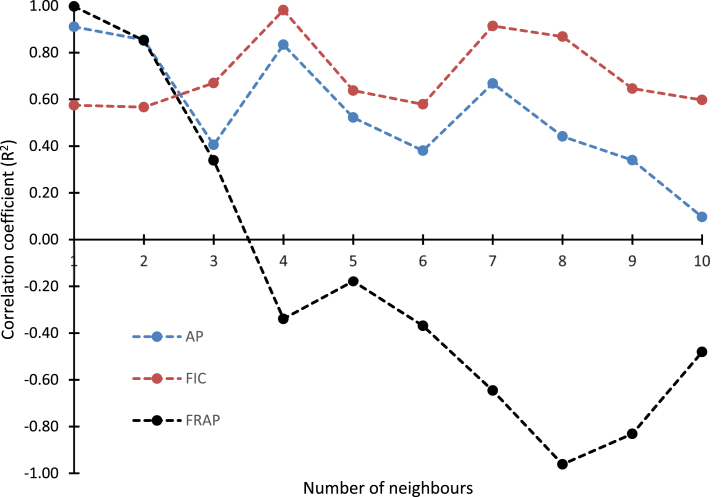
Plot for optimal numbers of neighbours.

Determinant. The effects of the number of neighbours on the correlation coefficient of the k-NN model are shown in Fig. (4). As shown in the figure, the increase in the number of neighbours resulted in a sinusoidal-like variation in the correlation coefficient. For antiradical power, the optimum number of neighbours at which the datasets were best predicted was 1 resulting in a correlation coefficient of 0.9104. When the number of neighbours was increased above one (1), there was overfitting which in turn resulted in poor prediction of the testing datasets. For ferrous ion chelating ability, the optimal n_neighbour where test accuracy was highest was found at n_neighbour = 4 which corresponded to a correlation coefficient of 0.9817. The increase in the number of neighbours in the prediction of the FRAP above one (1) led to significant overfitting of the testing datasets and hence, resulted in poor prediction of the datasets. Among the antioxidant activities, it could be seen that maximum ferric reducing power assay was obtained when the number of neighbours was within the n_neighbour range of 1–10. On the obtained number of neighbours where maximum correlation coefficient was obtained for the antioxidant activities, a k-NN model was used to predict the antioxidant activities of the seed extracts using the optimum number of neighbours and their predicted values are shown in Table 1.

### 3.6 Prediction of antioxidant activities of the seed extracts using decision tree (DT)

**Fig. 5:**
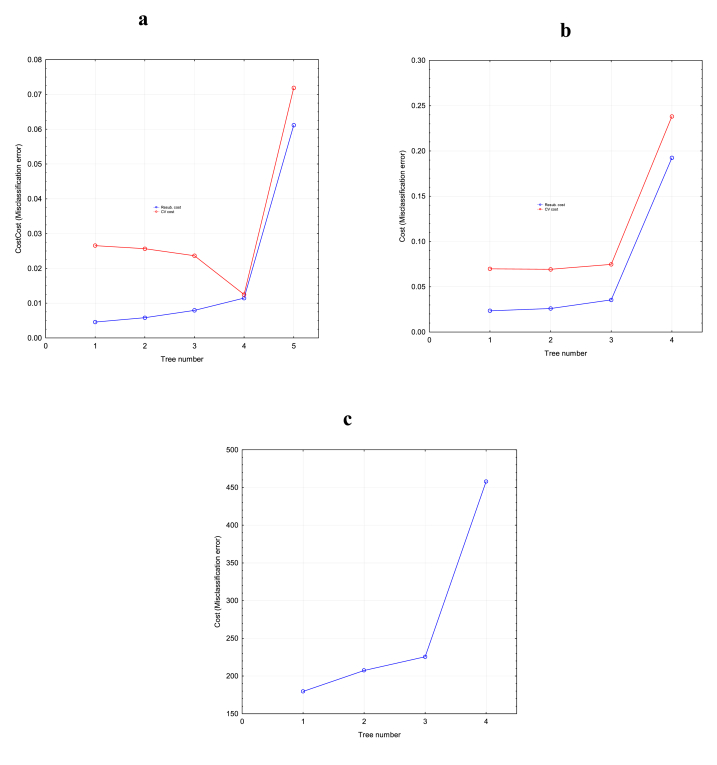
Selection of optimal decision tree based on resubstitution error rate and cross-validation error rate: (a) antiradical power; (b) ferrous ion chelating ability; (c) ferric reducing power assay.

For optimal prediction of the antioxidant activities of the seed extracts, three DT algorithms were used namely chi-squared automatic interactive detector (CHAID), classification and regression tree (CART) and exclusive CHAID were compared. Among these variants of DT algorithms, CART was adjudged to be the best predictor and a decision diagram was constructed using this algorithm. To model the antioxidant activities of the seed extracts using a decision tree, a 10-fold cross-validation method was used, and the results yielded a cross-validation rate which provided an evaluation of the predictive accuracy of the tree model at different tree sizes. The outcome of the cross-validation method is revealed in Fig. 5(a–c) which shows a plot of cross-validation error and resubstitution error rate against tree size. The resubstitution error describes the accuracy of the tree model in fitting the training datasets while the predictive ability of the tree model is well described by the cross-validation error. When a tree is large, the resubstitution error becomes smaller and hence, can cause overfitting. In this study, the optimal tree was selected based on minimum cross-validation error, and tolerance to resubstitution error, this was used to build the tree structure as shown in Fig. 6a, Fig. 6b, Fig. 6c below. The tree structure or graph for the antiradical power of the seed extracts is shown in Fig. 6a. From the tree, it could be seen that the predominant factor influencing the antiradical power of the seed extract was the solvent concentration. The tree consists of seven (7) nodes of which three (3) were non-terminal nodes while four (4) were terminal nodes. The root node (Node 1) is divided into two child nodes by solvent concentration. Node 2 consisted of 4 sample sizes with a solvent concentration ≤85 %, in which 0.46 % of the antiradical power was predicted. Node 3 with the sample size of eleven (11) extracts having a solvent concentration of >85 % predicted the antiradical power of the seed extracts at 0.96 %. Furthermore, the non-terminal Node 3 was divided into two subgroups based on extraction time (Node 6: ≤45 min; Node 7: >45 min) and the predicted antiradical power was 0.85 and 1 %, respectively. To predict the antiradical power of the seed extract from the top of the tree to the bottom along the branch of each leaf node of the tree, an “if-then" rule was generated and is shown below.

**Table:** 

Nodes	ARP (1/EC 50) (Pred)	Rules
Node 1	0.825	
Node 2	0.455	If B:Solvent concentration (%)≤85 then Antiradical Power (1/EC 50) = 0 in 26.7 % of cases
Node 3	0.959	If B:Solvent concentration (%) > 85 then Antiradical Power (1/EC 50) = 0 in 73.3 % of cases
Node 6	0.847	If B:Solvent concentration (%) > 85 and C:Extraction time (min)≤45 then Antiradical Power (1/EC 50) = 0 in 20 % of cases
Node 7	1.001	If B:Solvent concentration (%) > 85 and C:Extraction time (min) > 45 then Antiradical Power (1/EC 50) = 0 in 53.3 % of cases
Node 14	0.965	If B:Solvent concentration (%) > 85 and C:Extraction time (min) > 45 and A:Extraction temperature≤52.5 °C then Antiradical Power (1/EC 50) = 0 in 40 % of cases
Node 15	1.110	If B:Solvent concentration (%) > 85 and C:Extraction time (min) > 45 and A:Extraction temperature >52.5 °C then Antiradical Power (1/EC 50) = 0 in 13.3 % of cases

**Fig. 6a:**
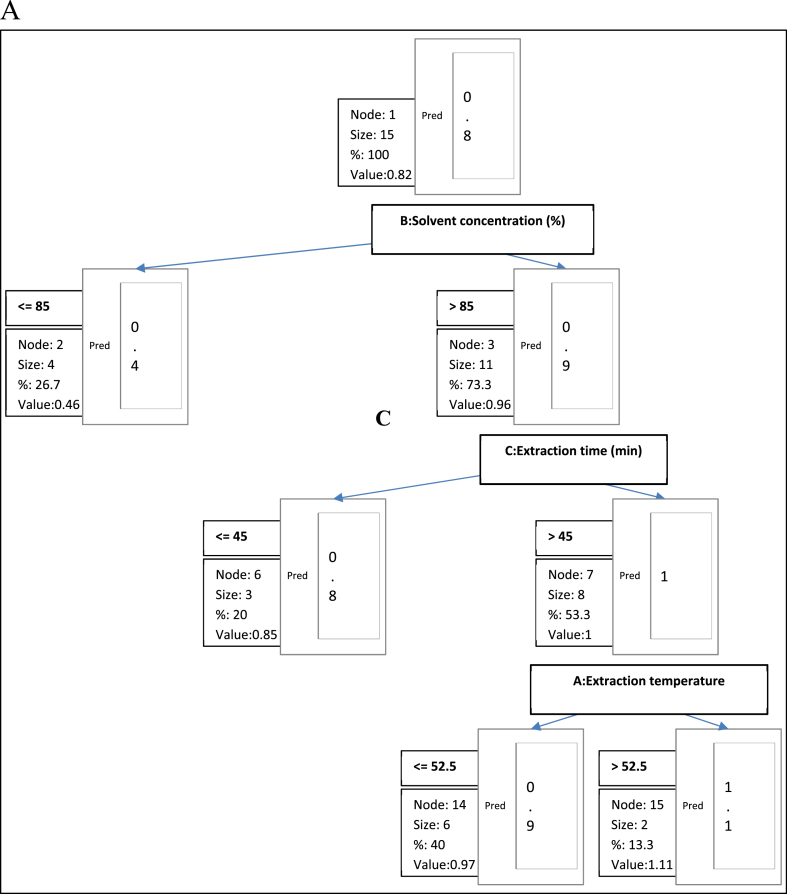
Decision tree diagram constructed by CHAID algorithm for the prediction of antiradical power.

**Fig. 6b:**
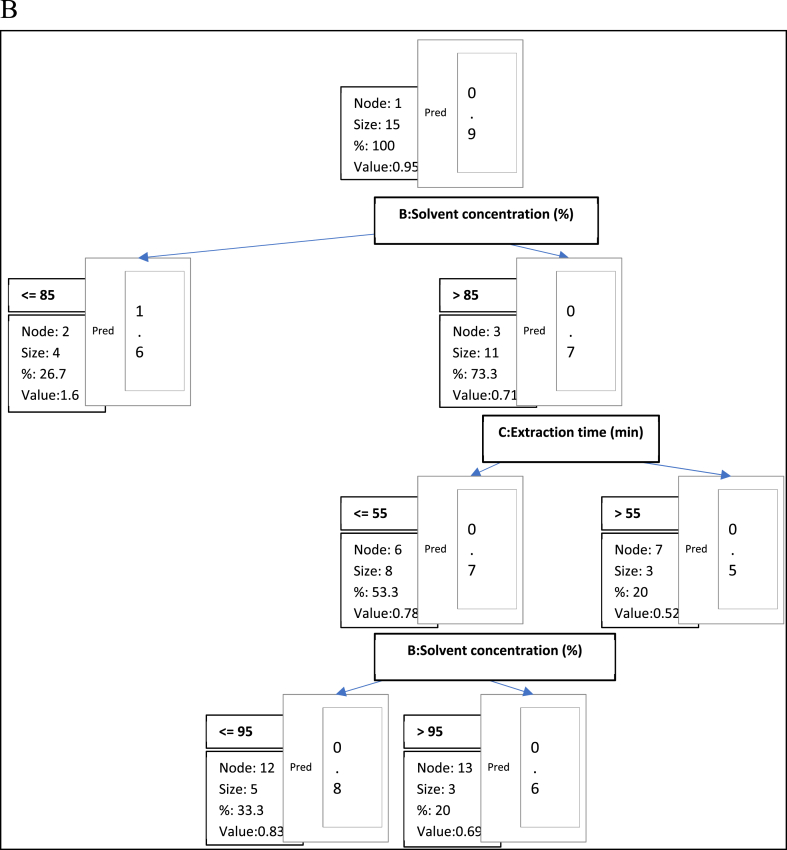
Decision tree diagram constructed by CHAID algorithm for the prediction of FIC.

**Fig. 6c:**
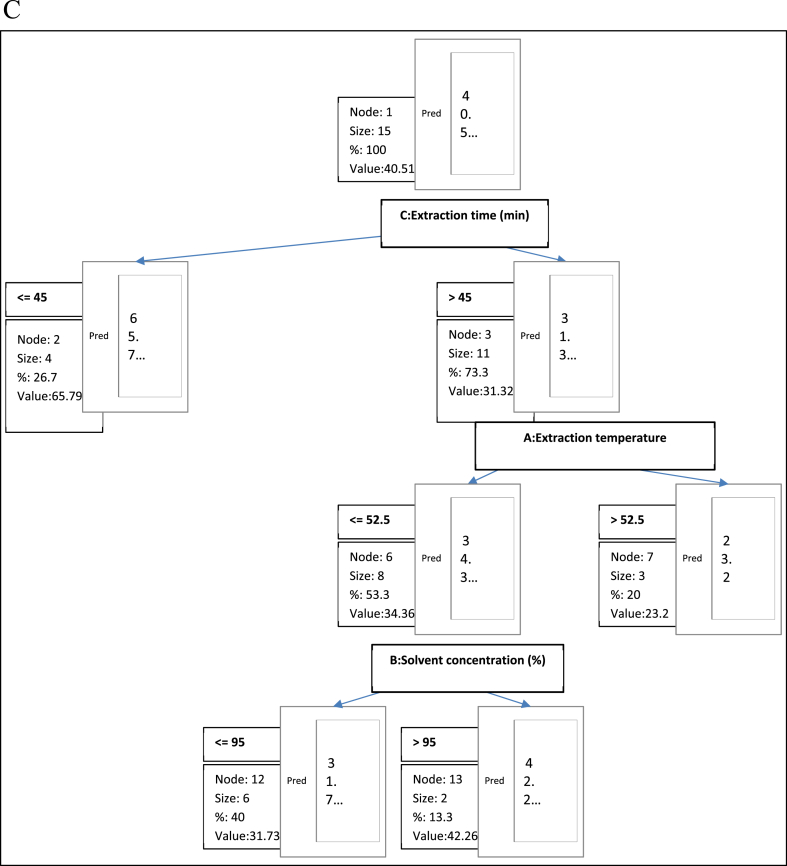
Decision tree diagram constructed by CHAID algorithm for the prediction of ferric reducing power assay.

In the decision tree for the ferrous ion chelating ability of the seed extracts (Fig. 6b), solvent concentration was the most important factor influencing the ferrous ion chelating ability. The nodes consist of 3 terminal nodes and 4 non-terminal nodes. The root node (Node 1) of the tree was divided into two child nodes (Node 2: ≤85 %; Node 3: >85 %). At the next stage of the tree structure and based on extraction time, Node 3 was divided into two subgroups (Node 6: ≤55 min; Node 7: >55 min). In this decision tree, the extraction temperature did not influence the ferrous ion chelating ability of the extracts. To predict the ferrous ion chelating ability of frap, the extract from the root node down to the bottom branch of the leaf nodes, an “if-then" rules were generated as stated below.

**Table:** 

Nodes	FCA (EC 50) (Pred)	Rules
Node 1	0.946	
Node 2	1.603	If B:Solvent concentration (%)≤85 then Ferrous ion Chelating (EC 50) = 0 in 26.7 % of cases
Node 3	0.707	If B:Solvent concentration (%) > 85 then Ferrous ion l Chelating (EC 50) = 0 in 73.3 % of cases
Node 6	0.777	If B: Solvent concentration (%) > 85 and C: Extraction time (min)≤55 then Ferrous ion Chelating (EC 50) = 0 in 53.3 % of cases
Node 7	0.521	If B: Solvent concentration (%) > 85 and C: Extraction time (min) > 55 then Ferrous ion Chelating (EC 50) = 0 in 20 % of cases
Node 12	0.831	If B:Solvent concentration (%) > 85 and C:Extraction time (min)≤55 and B:Solvent concentration (%)≤95 then Ferrous ion Chelating (EC 50) = 0 in 33.3 % of cases
Node 13	0.687	If B:Solvent concentration (%) > 85 and C:Extraction time (min)≤55 and B:Solvent concentration (%) > 95 then Ferrous ion Chelating (EC 50) = 0 in 20 % of cases

In terms of the ferric reducing power assay (Fig. 6c), the extraction time was predominantly the most important factor influencing the Frap of the seed extracts. The root node was divided into two child nodes at the time of extraction. Node 2 with an extraction time of ≤45 min consists of 4 samples size 4. The ferric reducing power assay in Node 2 was predicted as 65.79 %. Extracts obtained at extraction time >45 are composed in Node 3 while the frap predicted for extracts in this node was 31.32 %. The tree branches extend and Node 3 was further divided into two subgroups based on extraction temperature (Node 6: ≤52.5 with a frap prediction of 34.36 %; Node 7: >52.2 with a frap prediction of 23.2 %). The solvent concentration used in the extraction process is the least important factor influencing the FRAP content of the seed extract. An “if-then" rules was generated for the prediction of the Frap content of the seed extracts as stated below.

**Table:** 

Nodes	Frap (mg Fe(II)/100 g) (Pred)	Rules
Node 1	40.511	
Node 2	65.794	If C:Extraction time (min)≤45 then Frap (mg Fe(II)/100 g) = 0 in 26.7 % of cases
Node 3	31.317	If C:Extraction time (min) > 45 then Frap (mg Fe(II)/100 g) = 0 in 73.3 % of cases
Node 6	34.362	If C:Extraction time (min) > 45 and A:Extraction temperature≤52.5 then Frap (mg Fe(II)/100 g) = 0 in 53.3 % of cases
Node 7	23.196	If C:Extraction time (min) > 45 and A:Extraction temperature >52.5 then Frap (mg Fe(II)/100 g) = 0 in 20 % of cases
Node 12	31.730	If C:Extraction time (min) > 45 and A:Extraction temperature≤52.5 and B:Solvent concentration (%)≤95 then Frap (mg Fe(II)/100 g) = 0 in 40 % of cases
Node 13	42.259	If C:Extraction time (min) > 45 and A:Extraction temperature≤52.5 and B:Solvent concentration (%) > 95 then Frap (mg Fe(II)/100 g) = 0 in 13.3 % of cases

### 3.7 Performance evaluation of the machine learning algorithms

The performance evaluation of the machine learning algorithms based on benefit and cost performance metrics is shown in Table 4. The accuracy of the ML algorithms also known as the correct prediction ratio which is the total number of extracts in which their antioxidant activities were rightly predicted out of the total sample size ranged between 55.6 % and 86.7 %. Both artificial neural network and decision tree had the highest level of prediction accuracy while the accuracy of support vector machine regression to correctly predict the antioxidant properties of the seed extracts was low. The machine learning models were appraised for their predictability using R, R^2^, adjusted R^2^, SEP, MAE, RMSE and MSE. With respect to R values, the machine learning model ranked in the following ascending order SVM, k-NN, ANN and DT. The R-value close to 1 is an indication that there exists a high correlation between the actual and predicted values. The machine learning model on the other hand was ranked in decreasing order in terms of R^2^ and they are as follows; DT, ANN, k-NN and SVM. The R^2^ values of all the models are close to 1 which implies the extent to which the predicted antioxidants values can define the actual values when a linear relationship is desired between the predicted and actual values. The result obtained in this study is commensurate with the report of Zongur [24] who reported lower predictability of the SVM model over other machine learning models. Among the machine learning algorithms, it could be seen based on the performance metrics that the decision tree had the most predictive ability owing to its low root mean square of error (0.184), low mean square of error (0.0339), and highest.

**Table 4:** Performance evaluation of the machine learning algorithms.

**Parameters**	ANN	SVM	k-NN	DT
**Accuracy**	0.867	0.556	0.711	0.867
**R**	0.989	0.971	0.981	0.994
**R**^**2**^	0.980	0.943	0.963	0.988
**Adjusted R**^**2**^	0.975	0.930	0.955	0.985
**MSE**	0.034	0.174	0.284	0.034
**RMSE**	0.185	0.417	0.533	0.184
**MAE**	0.009	0.070	0.128	0.011
**AAD**	0.990	10.294	21.130	1.103
**SEP**	22.430	50.570	64.630	22.310

Coefficient of determinant (0.9878). To our knowledge, a study predicting the antioxidants of seed extracts using machine learning algorithms had not been studied previously.

## 4 Conclusion

In this study, the antioxidant activities of *Amaranthus viridis* seed extracts were predicted using four (4) machine learning algorithms namely artificial neural network, support vector machine-regression, k-nearest neighbour and decision tree. An extensive experiment based on a Box-Behnken design was performed to generate the dataset used in the training, testing and validation of the four data-driven models. Their performance and predictive ability were compared in terms of coefficient of correlation (R), coefficient of determinant (R^2^), adjusted R^2^, mean square error (MSE), root mean square error (RMSE), average absolute deviation (AAD) and mean absolute error (MAE). The extraction process was optimized using an artificial neural network coupled with a genetic algorithm. The result revealed that Decision tree model outperformed other machine learning models in predicting the antioxidant properties of *Amaranthus viridis* seed extracts with an accuracy of 0.867, coefficient of correlation of 0.9939 and correlation coefficient of 0.9878. It was closely followed by artificial neural network which was used to optimize and investigate the effects of extraction parameters on the antioxidant properties of the seed extracts. Optimizing the extraction condition with ANN-GA revealed a predicted antiradical power of 0.921 (1/Ec50), ferrous ion chelating ability of 0.719 EC_50_ and 87.31 mg Fe(II)/100 g extract. The obtained optimum extraction conditions were solvent concentration of 97.02 %, extraction time of 40 min and extraction temperature of 30 °C. This work thus demonstrated the predictability of antioxidant properties of the seed extracts using DT over other machine learning algorithms.

### 4.1 Limitation and future work

Owing to the modelling of the antioxidant properties of Amaranthus viridis seed extract using machine learning algorithms, there is a need to use other machine learning algorithms such as random forest, and naïve Bayes, as well as a deep learning approach for the prediction of the antioxidant properties of plant extracts. Also, owing to the flexibility of machine learning algorithms, there is a need for the usage of large data sets.

## Funding

The authors confirmed no funding was received for this research work.

## CRediT authorship contribution statement

**Babatunde Olawoye:** Conceptualization, Data curation, Formal analysis, Investigation, Methodology, Software, Writing – original draft, Writing – review & editing. **Oladapo Fisoye Fagbohun:** Methodology, Software, Writing – original draft. **Oyekemi Popoola-Akinola:** Investigation, Writing – original draft. **Jide Ebenezer Taiwo Akinsola:** Conceptualization, Software, Validation, Visualization, Writing – original draft. **Charles Taiwo Akanbi:** Supervision, Validation, Visualization, Writing – review & editing.

## Declaration of competing interest

The authors declare that they have no known competing financial interests or personal relationships that could have appeared to influence the work reported in this paper.
